# Proteostasis decline and endoplasmic reticulum stress in aging: Implications for cellular senescence and senescence-associated secretory phenotype regulation

**DOI:** 10.4103/NRR.NRR-D-25-00161

**Published:** 2025-08-13

**Authors:** Philippe Pihán, Lisa M. Ellerby, Claudio Hetz

**Affiliations:** Buck Institute for Research on Aging, Novato, CA, USA

Aging is a universal biological process characterized by the progressive decline in cellular and tissue function, representing the main risk factor for the development of most chronic human diseases. At the cellular level, one hallmark of aging is the accumulation of senescent cells—non-dividing yet metabolically active cells that adopt a unique phenotype, including the senescence-associated secretory phenotype (SASP) (Wang et al., 2024). The SASP encompasses a complex secretory program of bioactive molecules, including pro-inflammatory cytokines such as interleukin-6, interleukin-1 beta, and tumor necrosis factor-alpha; chemokines such as CXC motif chemokine ligand 8/interleukin-8 and C-C motif chemokine ligand 2; growth factors such as vascular endothelial growth factor and hepatocyte growth factor; and matrix-remodeling enzymes such as matrix metalloproteinases. These factors influence the surrounding microenvironment by promoting inflammation, tissue remodeling, and paracrine-induced senescence. While the SASP may play a beneficial role in acute stress responses such as wound healing and tumor suppression, its chronic persistence contributes to systemic inflammation, stem cell exhaustion, and age-associated pathologies (Wang et al., 2024).

Central to the aging process is the decline of the proteostasis capacity of cells, an interconnected network of pathways that ensure proper protein synthesis, folding, and degradation. Proteostasis is critical for maintaining a functional proteome under both normal and stress conditions. This network operates through three interconnected systems: molecular chaperones that facilitate protein folding and prevent aggregation, degradation pathways such as the ubiquitin-proteasome system and autophagy-lysosomal pathway that clear damaged proteins, and stress-responsive signaling mechanisms such as the unfolded protein response (UPR). Together, these systems maintain proteome integrity, ensuring cellular health.

With aging, proteostasis capacity diminishes. Studies across model organisms and human tissues demonstrate that molecular chaperones, degradation systems, and stress responses are less efficient in aged cells, leading to an accumulation of misfolded proteins and abnormal protein aggregates, impaired proteome integrity, and cellular dysfunction. In particular, the endoplasmic reticulum (ER)—a central organelle in protein folding—becomes increasingly burdened during aging (Hetz and Dillin, 2024). The ER relies on the UPR to manage its protein-folding load. This highly conserved pathway consists of three key branches: inositol-requiring enzyme 1 (IRE1), activating transcription factor 6 (ATF6), and protein kinase R-like ER kinase (PERK). IRE1α catalyzes the cytosolic splicing of a 26-nucleotide intron from the mRNA of X-box binding protein 1 (XBP1), altering its open reading frame and producing XBP1s (spliced), a potent transcription factor that drives the expression of numerous genes involved in proteostasis. Additionally, IRE1α mediates the degradation of several RNAs in a process known as regulated IRE1α-dependent decay (RIDD). ATF6, on the other hand, translocates to the Golgi apparatus, where it is cleaved by S1P and S2P proteases, generating ATF6f (fragment), another potent transcription factor that enhances the expression of chaperones and ER-associated degradation components. Under mild ER stress, the UPR restores proteostasis by attenuating protein translation, enhancing chaperone expression, and promoting the degradation of misfolded proteins. However, chronic or unresolved ER stress causes the UPR to transition from a protective mechanism to a pro-apoptotic signaling cascade, ultimately triggering cell death.

A critical component of the UPR is the PERK-eukaryotic initiation factor 2α (eIF2α) signaling axis, which plays dual roles in protein translation regulation and stress adaptation. Upon ER stress, PERK phosphorylates eIF2α, leading to transient inhibition of global protein synthesis. This translational pause reduces the folding burden of the ER while selectively enhancing the expression of stress-responsive genes such as ATF4, which upregulates genes involved in protein folding, metabolism, and apoptosis. Of note, eIF2α is also a central component of the integrated stress response, a broader cellular mechanism that responds to diverse stress signals. In addition to PERK, eIF2α can be phosphorylated by three other kinases: PKR (activated in response to viral infection), GCN2 (activated by amino acid deprivation), and HRI (activated by heme deficiency and the mitochondrial UPR). Together, these kinases integrate various stress signals, underscoring the critical role of eIF2α in cellular adaptation to stress.

Senescent cells exhibit high demands on their proteostasis network due to the secretion of SASP components, which include numerous proteins trafficked through the ER. The acquisition and maintenance of the SASP impose significant stress on the ER, which already contends with age-related declines in proteostasis capacity. Recent studies have shown that senescent cells exhibit increased basal expression of ER stress-associated proteins, such as the chaperone BiP and components of the ATF6 branch of the UPR, suggesting that these cells exhibit basal levels of ER stress (Hetz and Dillin, 2024). Additionally, multiple components of the UPR play a key role in the initial survival and establishment of the senescent state. For instance, IRE1α induces early senescence through RIDD of specific pro-oncogenic factors in Ras-induced models (Blazanin et al., 2017). This suggests that UPR signaling can serve as a barrier to tumorigenesis by promoting senescence in response to oncogenic stress. A model is emerging where, in aging, cells exhibit higher levels of basal ER stress while simultaneously displaying an inability to properly activate an adaptive UPR, leading to chronic, unresolved ER stress. However, the role of the UPR in regulating proteostasis and supporting the heightened secretory activity of senescent cells (i.e., SASP) under stress conditions remains poorly understood.

Recent findings by Payea et al. (2024) investigated how senescent cells respond to ER stress and proteostasis challenges. Using DNA-damaging agents such as etoposide and irradiation to induce senescence, they found that senescent cells display robust and persistent phosphorylation of eIF2α. However, despite this phosphorylation, translation of the downstream effector ATF4 is impaired, highlighting a decoupling of eIF2α phosphorylation from ATF4 translation (Payea et al., 2024). Mechanistically, this decoupling is linked to failures in ribosome availability and function. Ribosomal profiling revealed that senescent cells exhibit reduced translation activity and a significant reduction in ribosome numbers compared to cycling or quiescent cells. This reduction likely stems from decreased expression of ribosomal proteins, contributing to global translation inhibition in senescent cells.

The impaired adaptive response in senescent cells has significant consequences for their ability to cope with additional stressors. For instance, when treated with thapsigargin, a proteotoxic compound that induces ER stress, senescent cells fail to upregulate adaptive ER chaperones and stress-responsive proteins. This vulnerability highlights their inability to mount an effective UPR, suggesting that proteostasis dysfunction is a hallmark of senescence. Supporting these findings, a study in replicative senescent fibroblasts revealed that while basal expression levels of chaperones and proteostasis network components, as well as translation, were unchanged, senescent cells failed to upregulate chaperones and other stress-response components when subjected to heat shock stress (Sabath et al., 2020). Interestingly, under these conditions, senescent fibroblasts exhibited eIF2α phosphorylation coupled to ATF4 expression, indicating that stress responses may vary between different types of senescent cells. Together, these findings provide a molecular basis for the diminished stress resilience of senescent cells, which may exacerbate their contribution to aging and tissue dysfunction.

**Figure 1:**
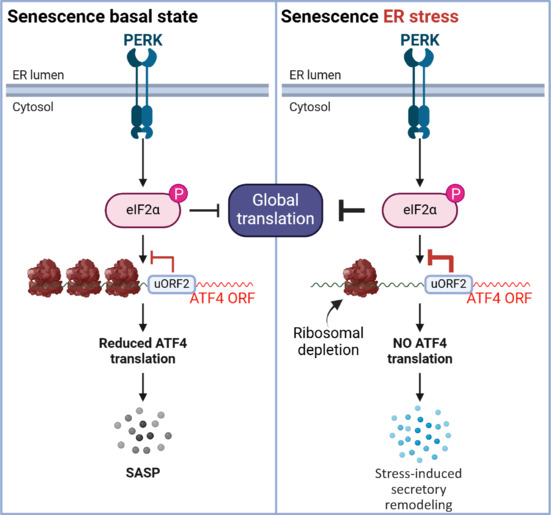
Ribosome dysfunction limits ER stress adaptation and promotes SASP remodeling in senescent cells. Under basal conditions, senescent cells exhibit persistent phosphorylation of eIF2α. However, ATF4 translation is impaired due to reduced ribosome availability and function, leading to global translational deficits. These impairments limit the ability of senescent cells to adapt to additional stressors. Right panel: Under ER stress conditions, senescent cells fail to upregulate adaptive ER chaperones and stress-responsive proteins. A global reduction in translating ribosomes and ribosomal proteins further enhances translation inhibition. Despite these deficits, senescent cells undergo stress-induced secretory remodeling, where SASP components are upregulated at the transcriptional level, amplifying their inflammatory phenotype. Created with BioRender.com. ATF4: Activating transcription factor 4; eIF2α: eukaryotic initiation factor 2 alpha; ER: endoplasmic reticulum; PERK: protein kinase RNA-like endoplasmic reticulum kinase; SASP: senescence-associated secretory phenotype; uORF: upstream open reading frame.

Interestingly, the consequences of proteostasis impairment extend to SASP regulation. Under ER stress conditions, many SASP components already present in senescent cells are further upregulated, increasing their expression even more, a phenomenon the authors termed stress-induced secretory remodeling (Payea et al., 2024). ER stress remodels the SASP in senescent cells by enhancing the transcription of secreted components, despite global translational deficits (**Figure 1**). Normally, ATF4 would play a critical role in resolving ER stress and attenuating the SASP. However, senescent cells are unable to efficiently upregulate ATF4 due to defects in ribosomal translation and ternary complex availability. This inability to resolve ER stress may exacerbate the inflammatory phenotype of the SASP, perpetuating a chronic state of non-homeostasis and amplifying the impact of senescent cells on tissue dysfunction and aging.

The implications of these findings may extend beyond cellular senescence to organismal aging. Impaired UPR signaling and proteostasis have been observed in aged tissues, correlating with increased senescence and systemic inflammation (Hetz and Dillin, 2024). For example, middle-aged and old mice treated with the ER stress inducer tunicamycin exhibit impaired activation of the IRE1α-XBP1s axis and increased hippocampal senescence. Conversely, enforced expression of XBP1s restores proteostasis and improves cognitive function, though it remains unclear whether these benefits arise directly from effects on senescence or broader restoration of proteostasis (Cabral-Miranda et al., 2022). Additionally, the PERK-eIF2α pathway is critical for metabolic control and glucose and lipid homeostasis. Heterozygous eIF2α mice exhibit reduced longevity, particularly in females, and develop metabolic syndrome, characterized by insulin resistance and disrupted lipid homeostasis (Anderson et al., 2021). It remains unclear whether these effects stem from the role of the UPR in cellular senescence or broader, undefined functions in other cell types during aging. The systemic nature of proteostasis decline further implicates inter-organ communication, where aging-associated ER stress in one tissue may propagate maladaptive signaling to others.

These recent findings by Payea et al. (2024) suggest that global translation inhibition and ribosomal depletion are hallmark features of DNA damage-induced senescence, limiting the capacity of senescent cells to mount adaptive responses to stress. At ER-mitochondria contact sites, localized inhibition of eIF2α phosphorylation has been observed, creating a translational “safe space” where mRNAs encoding mitochondrial proteins are selectively translated, even under conditions of global translation inhibition (Brar et al., 2024). This spatial regulation of translation suggests that ER-mitochondria contact sites may serve as key sites for sustaining mitochondrial function. Given that mitochondrial dysfunction is a hallmark of senescent cells, it will be important to investigate whether ER-mitochondria contact sites similarly regulate translation in senescent cells. Such studies could provide valuable insights into the biology of senescence and reveal novel therapeutic targets for modulating proteostasis in aging cells.

Therapeutic strategies targeting the proteostasis network hold significant promise for mitigating age-related dysfunctions. For example, ISRIB, a small molecule that counteracts the effects of eIF2α phosphorylation, has been shown to improve memory in aged mice, underscoring the essential role of protein translation in synaptic plasticity (Hetz and Dillin, 2024). Senomorphic drugs, which modulate the behavior of senescent cells by suppressing or remodeling their harmful secretory activity (SASP) without inducing cell death, provide an alternative to senolytics, which eliminate senescent cells through programmed cell death (Wang et al., 2024). The findings by Payea et al. (2024) demonstrate that the PERK-eIF2α axis influences the secretory phenotype of senescent cells under stress, highlighting the potential for UPR-targeting drugs to function as senomorphics by selectively modulating the SASP. Repurposing known UPR inhibitors and activators could offer an efficient strategy for modulating the SASP and cellular senescence. Such approaches may enable tailored interventions for specific senescent cell populations or tissues, providing a precise means of addressing aging-related pathologies.

Finally, the heterogeneity of senescence states poses significant challenges for therapeutic targeting. Different senescence-inducing stimuli activate distinct signaling pathways, and the role of the PERK-eIF2α axis may vary across senescent cell types. In senescent cells, maintaining low levels of ER stress is critical for survival, as the high demand on ER function from the SASP can otherwise lead to apoptosis. A genome-wide CRISPR screen revealed that inhibiting the YAP-TEAD pathway with verteporfin reduced senescent cell viability by inducing ER stress and apoptosis through mTOR inhibition. Verteporfin treatment also decreased senescent cell burden and improved tissue homeostasis in aging mice, highlighting an alternative senolytic strategy targeting ER activity required for SASP production (Anerillas et al., 2023). Another avenue for exploration is the relationship between the UPR, particularly the IRE1/RIDD pathway, and processes such as cell cycle arrest and the DNA damage response (Dufey et al., 2020). This pathway may play a significant role in the onset of cellular senescence. Further studies are needed to unravel the molecular mechanisms underlying the variability of senescent cells to stress responses and to identify context-specific interventions.

In conclusion, the decline of proteostasis and the dysregulation of the UPR are central to the pathophysiology of aging and cellular senescence. Senescent cells exhibit impaired stress responses and translation machinery dysfunction, which may arise from persistent DNA damage affecting cellular proteostasis. This disruption not only inhibits translation but also drives the remodeling of the SASP, amplifying its inflammatory effects and further compromising tissue homeostasis. Targeting the proteostasis network offers a promising strategy to alleviate the consequences of aging and senescence. However, the complexity and heterogeneity of these processes necessitate further investigation to develop precise and effective interventions. A deeper understanding of the intricate relationship between proteostasis, DNA damage, and the SASP could transform approaches to age-related diseases and improve healthy lifespan.

*This work was supported by NIH NIA 1RO1AG061879 and 5PO1AG066591 (to LME) and FONDAP Program 15150012, ECOS-ANID (ECOS230034), and the US Army Medical Research Acquisition Activity (USAMRAA) AL2201415 (to CH).*
